# Effects of Sucralose Supplementation on Glycemic Response, Appetite, and Gut Microbiota in Subjects with Overweight or Obesity: A Randomized Crossover Study Protocol

**DOI:** 10.3390/mps7050080

**Published:** 2024-10-07

**Authors:** Zeniff Reyes-López, Viridiana Olvera-Hernández, Meztli Ramos-García, José D. Méndez, Crystell G. Guzmán-Priego, Miriam C. Martínez-López, Carlos García-Vázquez, Carina S. Alvarez-Villagomez, Isela E. Juárez-Rojop, Juan C. Díaz-Zagoya, Jorge L. Ble-Castillo

**Affiliations:** 1 Centro de Investigación, División Académica de Ciencias de la Salud (DACS), Universidad Juárez Autónoma de Tabasco (UJAT), Villahermosa 86150, Mexico; 2 Hospital de Cardiología, Centro Médico Nacional Siglo XXI, Instituto Mexicano del Seguro Social (IMSS), Ciudad de México 06703, Mexico; 3 División Académica de Ciencias Biológicas (DACBIOL), Universidad Juárez Autónoma de Tabasco (UJAT), Villahermosa 86150, Mexico; 4 División de Investigación, Departamento de Bioquímica, Facultad de Medicina, Universidad Nacional Autónoma de México (UNAM), Ciudad de México 04510, Mexico

**Keywords:** sucralose, non-nutritive sweeteners, glycemic response, appetite, gut microbiota, crossover study, obesity

## Abstract

Sucralose stands as the most common non-nutritive sweetener; however, its metabolic effects have sparked significant controversy over the years. We aim to examine the effects of sucralose daily intake on glycemia, subjective appetite, and gut microbiota (GM) changes in subjects with overweight or obesity. In this randomized, crossover, and controlled trial, 23 participants with a body mass index between 25 kg/m^2^ and 39.9 kg/m^2^ will be assigned to one of two interventions to receive either sucralose (2 mg/kg/day equivalent to 40% of the acceptable daily intake) or glucose (control) for 4 weeks, each phase separated by a 4-week washout period. The glycemic response will be determined during a meal tolerance test, subjective appetite will be evaluated using a visual analog scale, and GM changes will be analyzed by next-generation sequencing of the bacterial rRNA 16S gene from fecal samples. All measures will be performed before and after intervention periods. We hypothesize that sucralose supplementation induces changes in glycemic response, subjective appetite, and gut microbiota in overweight and obese participants. This protocol was approved by the Ethics Committee of the UJAT (No. 0721) and was registered in the Australian New Zealand Clinical Trials Registry (ACTRN12621001531808).

## 1. Introduction

Global obesity and overweight rates have risen sharply, becoming a major health concern due to their association with type 2 diabetes (T2D) and cardiovascular disease. Mexico ranks second in obesity prevalence among the nations belonging to the Organization for Economic Cooperation and Development (OECD), with 36.9% of its population affected [1]. Non-nutritive sweeteners (NNS) have been implemented as substitutes for sugar to reduce caloric intake and prevent body weight gain. NNS can be artificially produced as sucralose, aspartame, acesulfame K, or saccharin or can be obtained from natural sources, such as steviol glycosides or monk fruit [2].

Sucralose is an artificial NNS derived from sucrose, which has a sweetening capacity 600 times greater than sucrose and has an acceptable daily intake (ADI) estimated value of 5 mg/kg/day according to the Food and Drug Administration (FDA) [3]. The use of sucralose is widely recommended among individuals with obesity and T2D. However, experimental and clinical trials have reported undesirable effects on glucose and energy metabolism, which can be attributed to three potential mechanisms: activation of T1R2/T1R3 sweet taste receptors, affecting insulin and incretin release; triggering a cephalic response that may increase caloric intake; and interaction with gut microbiota (GM), potentially causing dysbiosis. In the latter case, sucralose may disrupt GM composition, increasing intestinal permeability and allowing lipopolysaccharides (LPS) to enter the bloodstream, leading to a low-grade inflammatory response (metabolic endotoxemia) that could induce insulin resistance [4].

In humans, the effects of realistic doses of sucralose on glucose metabolism have been mostly assessed in healthy subjects. Suez et al. showed a clear alteration in glycemic response after two weeks of consuming sucralose (1.8 mg/kg BW/day equivalent to 34% ADI, FDA), which was correlated with GM alterations [5]. Romo-Romo et al. reported an increase in insulin resistance estimated by the HOMA-IR index after only two weeks of sucralose consumption (2.25 mg/kg BW/day equivalent to 45% ADI, FDA) [6]. Recently, Mendez-García et al. (2022) found an altered glycemic response related to changes in GM composition after sucralose intake (0.65 mg/kg BW/day equivalent to 13% ADI, FDA) for 10 weeks [7]. Conversely, other studies in humans do not identify the harmful effects of sucralose on glycemic response or GM [2,8,9]. Thus, it is unclear whether sucralose can induce metabolic alterations modulated by the microbiota modifications.

Particularly, most previous studies examining glycemic response and appetite modifications to sucralose administration have been largely limited to studies in animal models or individuals with normal body weight [10,11,12,13,14,15,16,17]. However, subjects with increased body weight represent an important group due to their increased risk of developing metabolic diseases such as diabetes, hypertension, and heart disease. For this reason, we will conduct a crossover, randomized, and controlled study in subjects with overweight or obesity. The primary outcome of the study will be the glycemic and insulinemic responses, and the secondary outcomes will include pre-post intervention changes in fasting parameters of glucose, insulin, total cholesterol (TC), high-density lipoprotein cholesterol (HDL-C), and triglycerides. Additionally, pre-post changes in anthropometric measures, blood pressure, GM composition, and subjective appetite will be considered. The knowledge gained in this study about the potential metabolic alterations induced by sucralose will be crucial in guiding recommendations for its use, especially given its widespread presence in the food industry.

## 2. Experimental Design

### 2.1. Participants

A total of 23 adults of both sexes will be recruited. During the screening process, subjects will assist with an interview with a healthcare provider to determine anthropometric indexes, blood pressure, fasting glucose determination, and medical check-ups. Participants will fulfill the following inclusion criteria: both genders, aged 18 to 50, overweight or obese (25–39.9 kg/m^2^), with fasting glucose < 125 mg/dl, and with a maintained stable weight in the previous three months. Patients receiving antibiotics, probiotics, or prebiotics without the ability to give informed consent, pregnant or breastfeeding women, smokers, or alcoholics who have allergies to sucralose or food intolerance, and those with heart, liver, gastrointestinal, or renal disorders will not be included. Patients who have sensitivity or discomfort related to the high sweetness of the test beverage and patients who need to receive treatments with antibiotics, probiotics, or other treatments with drugs that interfere with carbohydrate metabolism or microbiota integrity during the experimental period will be excluded.

### 2.2. Interventions

A dose of 2 mg/kg/day of commercial sucralose (Golden Hills) will be administered to participants in the sucralose intervention arm for 4 weeks. This dose is equivalent to 40% of the acceptable daily intake (5 mg/kg/day, FDA). Sucralose will be provided to participants in commercial sachets for mixing into their habitual beverages, to be consumed with their three main daily meals. The sucralose dose will be adjusted according to the body weight using an Excel fact sheet based on its concentration in the sachets (2% m/m in glucose). The control group will receive only pure glucose (dextrose), according to the amount of glucose present in the sachets administered in the sucralose arm. The glucose will be weighed and provided to the patients in separate sachets. Adherence will be monitored by personal phone calls and messages via WhatsApp; also, a daily food record format will be given to the participants to provide the number of sachets consumed during the test days. Later, low-NNS intake will remain during the experiment, according to the researcher’s recommendations.

### 2.3. Meal Tolerance Test

A 2 h MTT will be performed before and after each intervention phase to assess glycemic and insulinemic responses, as well as subjective appetite. Prior to the MTT day, it will be requested that subjects not consume alcohol and do not perform intense exercise during 24 h. On test days, after 12 h fasting, subjects will be appointed to the research center at 7:00 a.m., and they will first complete a 100 mm visual analog scale (VAS) self-reported paper questionnaire (time point 0) to measure subjective appetite [18]. Then, an intravenous catheter will be inserted into the antecubital vein of each subject, and a fasting blood sample will be taken (time point 0) for biochemical determinations. After, subjects will be given a standardized breakfast that consists of a nutritional supplement (Simisure; Modern Strategies; Mexico) and a 22 g Kellogg’s Rice Krispies bar (Kellogg’s Company, Battle Creek, MI, USA). The breakfast’s nutritional distribution will be added up to a total of 210 kcal, 7 g from protein, 38.5 g from carbohydrates, and 3 g from lipids. Blood samples will be collected after this breakfast at 15, 30, 60, 90, and 120 min. Glucose and insulin concentrations will be determined in these blood samples, and the VAS questionnaire will be applied 5 min before each sampling. During the test period, subjects will stay at the research center and will be free to read, use electronic devices, watch television, or engage in conversation.

### 2.4. Subjective Appetite

To assess appetite sensation, a validated VAS will be administered before and after each intervention phase. VAS consists of 100-mm-long lines. Each line´s endpoints are labeled with words indicating the most intense positive and negative appetite sensations. Hunger, satiety, fullness, and prospective food consumption will be assessed. Questions will be asked as follows: (1) How hungry do you feel? (2) How satisfied do you feel? (3) How full do you feel? (4) How much food do you think you could eat?

### 2.5. Anthropometric Measurements and Blood Pressure

Body weight and composition will be measured using a body composition analyzer based on bioelectrical impedance (Tanita, TBF-300A, Mexico City, Mexico). Height will be measured using a portable stadiometer (Seca^®^ 214). BMI will be determined using weight and height measurements, and the classification will be made following the World Health Organization (WHO) guidelines: underweight (<18.5 kg/m^2^), normal (18.5–24.9 kg/m^2^), overweight (25–29.9 kg/m^2^), and obesity (30.0 kg/m^2^). A retractable non-stretching tape measure will be used for waist circumference (WC) and hip circumference (HC) measurements. WC will be measured halfway between the bottom rib and iliac crest, while HC will be taken at the widest part of the buttocks. These measurements will determine the waist-hip ratio. Blood pressure will be measured twice using an Omron HEM-7120 professional portable blood pressure monitor. The two measurements of the blood pressure will be taken within one minute, as recommended in the Clinical Practice Guidelines on the Management of Hypertension [19]. The final reading will be based on the average of two readings recorded. Both the anthropometric and blood pressure measurements will be taken before and after each intervention phase.

### 2.6. Biochemical Determinations

Blood samples from the antecubital vein will be collected before and after each intervention phase to determine glucose, insulin, triglycerides, TC, and HDL-C. Serum samples that will not be immediately analyzed will be stored at −70 °C until further analysis. Glucose, cholesterol, HDL-C, and triglyceride determinations will be performed using a Spinreact, Spin200E Clinical Chemistry Autoanalyzer. Insulin will be measured by chemiluminescent microparticle immunoassay assay (CMIA) using a Wiener Lab CLIA-900 System, Rosario, Argentine. To estimate insulin resistance (IR), the homeostatic model assessment index (HOMA-IR) will be used. This is calculated by multiplying fasting glucose (mg/dL) and insulin (μU/mL) concentrations, then dividing by 405 [20].

### 2.7. Gut Microbiota Composition

The fecal samples will be collected for gut microbiota analysis before and after each intervention phase. Microbial DNA extraction from fecal samples will be performed using the QIAamp^®^ Fast DNA Stool Mini Kit (QIAGEN, Hilden, GE, USA) (Cat No. 51604) according to the manufacturer’s instructions. DNA concentrations will be measured with the Nanodrop 2000 (Thermo Fisher Scientific, Waltham, MA, USA). The 16S ribosomal RNA (rRNA) gene will be amplified by polymerase chain reaction (PCR). The PCR products will be visualized using agarose gels.

## 3. Procedure

### 3.1. Ethical Aspects

Research will be conducted in accordance with the principles of the Declaration of Helsinki. The study protocol was approved on 22 April 2021 by the Ethics Committee of the Universidad Juárez Autónoma de Tabasco (UJAT) (No. 0724), and it was registered on 10 April 2021 at the Australian New Zealand Clinical Trials Registry (ACTRN12621001531808). This trial will take place at the División Académica de Ciencias de la Salud (DACS) at UJAT. Informed consent will be obtained from participants by trained research coordinators., who will explain the study’s aims, potential risks, and participants’ right to withdraw from the experiment at any time. Personal information from enrolled participants will be collected and stored confidentially. As part of the informed consent process, participants will be informed that the study protocol includes the collection of blood and fecal samples.

### 3.2. Trial Design

**Figure 1:**
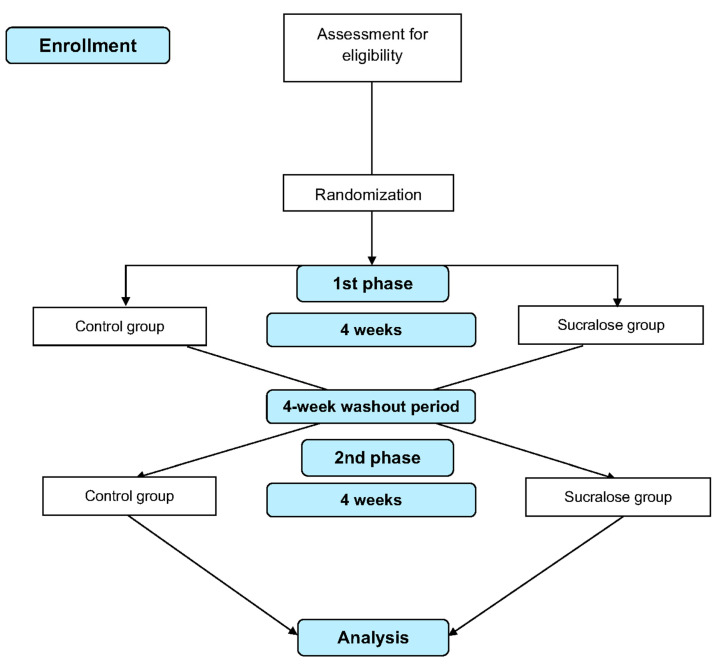
Flow diagram of the study design.

This study is a crossover, randomized controlled trial (RCT) to determine the effect of daily intake of sucralose on glycemic response, subjective appetite, and GM in subjects with overweight or obesity. Participants will be invited to attend the laboratory for preliminary measurements and baseline data after providing written informed consent. Following this, they will be randomly allocated into one of two treatment groups to receive either sucralose or glucose (control) daily for four weeks, each phase separated by a four-week washout period. An MTT, subjective appetite assessment, and stool collection will be carried out before and after each intervention phase (four measurement points). A flow diagram of the study protocol is shown in Figure 1, and the participant timeline actions are presented in Table 1.

### 3.3. Recruitment

Trial participants who are overweight and obese will be screened and recruited by researchers at the DACS of the UJAT from Villahermosa, Tabasco, Mexico. Moreover, information flyers via social media containing the details of the trial will be posted at respective institutions, hospitals, and other public places. The researchers will enroll patients by the inclusion and exclusion criteria. Patient recruitment started in September 2023. A computer-based online random number generator (www.random.org) will be used to allocate participants to the respective interventions, either sucralose or glucose. Randomized treatments will be supplied in sealed envelopes.

### 3.4. Safety Assessment

Each participant will be assessed for any adverse events during the clinical study. Sucralose consumption may cause mild discomfort related to its high sweetness power; however, this will depend on the sensitivity of the participant. At the site of the venous puncture, a bruise and/or a sensation of mild pain may appear. Some unexpected side reactions may appear, which vary in each subject, and these may not necessarily be caused by the treatments. In case of discomfort or adverse effects, participants will be treated by a healthcare provider.

### 3.5. Data Collection and Management

The research staff will interview subjects to gather demographic information and other baseline characteristics of the study group, including NNS consumption frequency, anthropometric measures, and medical history (comorbidities, medications, smoking). All participants will receive medical and nutritional sessions during and following the study. At no time will the participants be identified by the publications or presentations derived from this study, and each of the data provided will be handled with absolute confidentiality. Stool samples will be used exclusively for genetic analysis of the bacteria. The genetic information of the participants will not be studied or analyzed.

### 3.6. Statistical Analysis

Sample size calculation was performed using NCSS and PASS v. 11.0 software. To detect a difference of 10% in the primary outcome variable (glycemic response AUC) and considering a power of 80% and a type 1 error of 5% in a crossover design, a sample size of 19 participants was calculated. However, to allow for a dropout rate of 20%, 24 participants will be considered. Four samples per participant will be analyzed: two baselines and two endpoints. The primary analysis will be performed using GraphPad Prism v. 7.0 and QIIME Software. The D’Agostino and Pearson tests will be used to determine Gaussian distributions. Differences in fasting biochemical markers will be assessed using a one-way analysis of variance (ANOVA) in combination with Tukey’s post hoc test. Time-course data will be analyzed by two-way repeated measures (RM) ANOVA to assess the effects of treatment, time, and the interaction of treatment and time. The intervention´s effect on GM community dissimilarity will be analyzed with permutational multivariate analysis of variance (PERMANOVA) on the Bray–Curtis distance and visualized with Principal Component Analysis (PCoA). ANOVA, or the Kruskal–Wallis test, will be used to compare alpha diversity using the Shannon index. The Firmicutes/Bacteroidetes ratio (F/B ratio) will be calculated from relative abundances. A *p*-value < 0.05 will be considered significant for all analyses.

## 4. Expected Results

The present study will investigate the effects of realistic doses of sucralose (40% ADI) on metabolic markers, appetite, and GM changes in subjects with overweight or obesity. The problem of obesity has spread globally, not just confined to developed countries. Over the past decades, there have been predictions that if the current trend continues, most of the adult population worldwide will be overweight or obese by 2030 [21]. In this context, non-nutritive sweeteners have been implemented to lower calorie consumption and, consequently, mitigate the risk of increased body weight [22]. However, some studies have demonstrated its participation in the development of metabolic alterations.

Some studies have focused on assessing the effects of sucralose on fasting glucose or glycemic and insulinemic responses in animal models [14,23,24,25,26,27] and in healthy or with T1D/T2D subjects [2,6,7,9,13,15,28,29,30,31]. Other studies show evidence of changes in appetite parameters from a single-dose consuming sucralose [10,12,17,32,33,34]. However, the limited evidence evaluating the effects of sucralose consumption on metabolic markers, appetite, and GM in overweight or obese subjects is inconclusive [35,36,37,38]. This study aims to provide crucial information to this population by evaluating some different factors that have been separately studied under varying conditions, mainly in healthy individuals. This planned study has several strengths. First, notably, this is an RCT that will evaluate metabolic markers and appetite associated with GM composition as an explanatory variable in subjects with overweight or obesity. Second, a crossover trial will be used to minimize inter-individual variability since each participant acts as his own control. One advantage of this design is that fewer participants compared to parallel trials are needed to achieve the same level of significance in terms of type I and II errors. Third, this study involves realistic doses of sucralose. Fourth, this is a long-term study, as the interventions will consist of four weeks, separated by a four-week washout period. Overall, the findings of this study will assist health professionals in making more informed recommendations regarding the consumption of commercial sweeteners and food products containing sucralose. 

## Figures and Tables

**Table 1:** Schedule of enrollment, interventions, and assessments, according to the SPIRIT 2013 statement. During the intervention periods, all the assessments were performed a day before and after interventions in the respective phases.

**TIMEPOINT (Weeks)**	**STUDY PERIOD**										
	**Enrolment**	**Allocation**	**Phase I**				Wash-out period (4 Wks)	**Phase II**			
			**Wk 1**	**Wk 2**	**Wk 3**	**Wk 4**		**Wk 1**	**Wk 2**	**Wk 3**	**Wk 4**
ENROLLMENT:											
Eligibility screen	x										
Informed consent	x										
Demographic	x										
Medical history	x										
Anthropometric measurements	x										
Blood pressure	x										
Clinical blood sampling	x										
Allocation		x									
INTERVENTIONS:											
Sucralose			x	x	x	x		x	x	x	x
Control			x	x	x	x		x	x	x	x
ASSESSMENTS:											
Meal Tolerance Test			x			x		x			x
Appetite measurement			x			x		x			x
Fasting Blood sampling	x		x			x		x			x
Stool Sample Collection			x			x		x			x
Blood Pressure	x		x			x		x			x
Anthropometric measurements	x		x			x		x			x

## Data Availability

No new data were created or analyzed in the study.
